# 2D and 3D Models of Alzheimer’s Disease: Investigating Neuron-like Cells in Oxidative Environments

**DOI:** 10.1021/acsomega.5c03306

**Published:** 2025-06-16

**Authors:** Geisa R. Salles, Luiza A. Giraldi, Newton S. da Silva, Marimelia A. Porcionatto, Cristina Pacheco-Soares

**Affiliations:** 1 Research & Development Institute, 67655 Universidade do Vale do Paraíba, Av. Shishima Hifumi, 2911, Urbanova, São José dos Campos 12244-000, Brazil; 2 Universidade Estadual Paulista, Institute of Science and Technology, Av. Eng. Francisco José Longo, 777, Jardim São Dimas, São José dos Campos 12245-000, Brazil; 3 Department of Biochemistry, Universidade Federal de São Paulo, Escola Paulista de Medicina, R.Pedro de Toledo, 669, Vila Clementino, São Paulo 04039-032, Brazil; 4 National Institute of Science and Technology in Modeling Human Complex Diseases with 3D Platforms (INCT Model 3D), R.Pedro de Toledo, 669, Vila Clementino, São Paulo 04039-032, Brazil

## Abstract

Alzheimer’s disease (AD) is a complex and enigmatic neurodegenerative disorder in which amyloid-β (Aβ) aggregates and oxidative stress play crucial roles in neuronal damage. Aβ forms senile plaques, while reactive oxygen species (ROS)-induced oxidative stress causes cellular dysfunction. Elucidating neuronal injury led by mild and severe oxidative stress may provide insight into how neurons respond to toxic environments. In parallel, modeling AD three-dimensionally is in the spotlight, sustainably contributing to reducing animals in research and replicating spatially neuronal mechanisms, such as neurite network and oxidative stress responses. This study evaluates the effects of oxidative stress on neuron-like cells cultured in two-dimensional (2D) and 3D spheroids, strengthening their potential as platforms for AD investigation. For the 2D models, SH-SY5Y (cells from human neuroblastoma) cells were differentiated into the neuronal phenotype and exposed to mild or severe concentrations of oxygen peroxide (H_2_O_2_, 100 or 200 μM, respectively). Cytoviability, ROS, Aβ particle analyses, and morphological aspects were assessed. Neuronal cells under severe stress produced elevated levels of intra- and extracellular Aβ aggregates. A range of Aβ particle analyses were performed comparing their properties, and morphologically, neurites were compromised under severe stress. For the 3D models, SH-SY5Y spheroids were self-assembled by 10 days of cultivation on developed nonadhesive hydrogel microwells and differentiated into the neuronal phenotype; their area, circularity, and solidity were measured. Spheroids were exposed or not to 200 μM H_2_O_2_, stained for cytoskeleton/nuclei, and imaged by scanning electron microscopy (SEM), and their viability was evaluated. Throughout the cultivation period, spheroids grew and differentiated morphologically. Neurite distribution was observed along the 3D composition; however, under oxidative stress, cytoviability decreased, abnormal nuclear staining was observed surrounding the spheroids, and morphological disorganization was evident by SEM, representing structural disarrangement and nuclear disruption. Briefly, this study provides a basis for exploring oxidative stress and producing robust 3D approaches to unraveling AD mechanisms.

## Graphical abstract

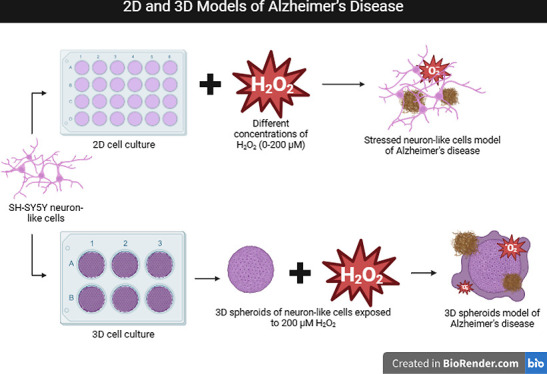

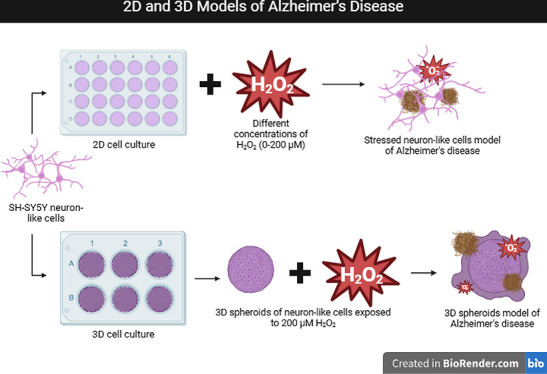

## 1 Introduction

Alzheimer’s disease (AD), the predominant cause of dementia globally, is characterized by progressive cognitive decline and distinct neuropathological features, including amyloid-beta (Aβ) senile plaques, tau neurofibrillary tangles, heightened oxidative stress, and neuronal loss.^1^ Despite extensive research, the underlying mechanisms of AD pathogenesis remain insufficiently understood. To address this knowledge gap, there is a critical need for advanced experimental models that can accurately replicate the cellular and molecular characteristics of the disease.

Oxidative stress, characterized by an imbalance between the production of reactive oxygen species (ROS) and antioxidant defenses, is a well-established contributor to the pathogenesis of AD.^2–4^ Elevated ROS levels in AD lead to oxidative damage of lipids, proteins, and nucleic acids, thereby exacerbating synaptic dysfunction, mitochondrial impairment, and neuronal death. Crucially, oxidative stress promotes the production, aggregation, and accumulation of Aβ peptides, which in turn further exacerbate oxidative stress, resulting in a cycle that drives neurodegeneration.^5–7^

The pathological interplay between oxidative stress and Aβ^8^ impacts various bodily functions, depending on the severity of AD. Under mild conditions, AD patients may retain the ability to perform daily activities such as working and driving, with occasional support. At this stage, Aβ plaques gradually accumulate, disrupting neuronal communication and leading to mild cognitive impairments. Moreover, there are early synaptic disfunction^9^ and reduced glucose uptake into the brain.^10,11^ Oxidative stress also plays a crucial role during this stage, leading to the production of ROS, causing further damage to neurons and synaptic connections.^12–14^ Unfortunately, AD is progressive and neurodegenerative; as the disease advances, increased neuronal damage and widespread brain atrophy enhance cognitive and functional impairments. Under severe conditions, the role of oxidative stress becomes more pronounced, exacerbating neuronal damage and contributing to the overall neurodegenerative process. Excessive senile plaques of Aβ disrupt neuronal communication and increase oxidative stress and mitochondrial dysfunction. Ultimately, increased inflammation and cell death further exacerbate the condition, and the patient necessitates comprehensive care and support.^15^

This synergy between oxidative stress^16^ and Aβ aggregates^17^ along the stages of AD represents a key element in the progression of the disease and highlights the necessity of elucidating the characteristics of Aβ aggregates produced under varying intensities of oxidative stress by brain cells.

In parallel, three-dimensional (3D) models, such as neuronal spheroids, have emerged as a promising platform for studying complex neurodegenerative diseases like AD.^18,19^ These models replicate the spatial conditions of the brain, including cell–cell, cell–protein, and cell–matrix interactions, exploring the cross talk between cells and even systems.^20,21^ They are particularly advantageous for investigating AD, as the effects of oxidative stress and extracellular Aβ aggregates on neuronal health can be observed three-dimensionally, providing a more accurate resemblance to the pathology. Additionally, they allow for precise spatial neurite distribution, creating a more relevant system for studying disease progression and therapeutic responses, where cells interact multidimensionally, similar to *in vivo* conditions.

Given the pivotal role of oxidative stress in AD, this study conducted a detailed analysis of cellular events and Aβ particles produced by neuronal cells under mild and severe oxidatively stressed environments. Moreover, we developed a 3D neuronal spheroid model subjected to oxidative stress. These models represent powerful tools for investigating therapeutic applications and contribute to elucidating the complex cellular dynamics and pathways involved in AD.

## 2 Materials and Methods

### 2.1 Study Design

This study was designed to develop 2D and 3D models of oxidative stress in human neuron-like cells, resembling this classic hallmark of AD. Briefly, SH-SY5Y cells (human neuroblastoma cells) were cultured in a monolayer (2D models), or self-assembly spheroids were made in nonattachable agarose-based microwells (3D models). Cells were differentiated into neuronal phenotypes and stressed with H_2_O_2_. Spheroids were characterized, and biological aspects of both 2D and 3D models were evaluated, including cytoviability, Aβ aggregates (stained area and particle analyses), neurite distribution, ROS, and morphology.

### 2.2 Reagents and Equipment

All reagents and equipment used in this study are described in Table 1.

**Table 1:** Reagents and Equipment

reagent or resource	source	identifier
reagents, antibodies, supplements, and other chemicals		
2′,7′-dichlorodihydrofluorescein diacetate (DCFDA)	Sigma-Aldrich, USA	287810
acetone	Emsure, Germany	1.00014.1000
agarose	Sigma-Aldrich, USA	A9539
CellROX (orange reagent)	Invitrogen, USA	C10443
Congo red	Neon, Brazil	01551-DSYS
DAPI	Sigma-Aldrich, USA	D9564
Dulbecco’s modified Eagle’s medium/nutrient mixture F-12 (DMEM/F12)	Gibco, USA	12400024
fetal bovine serum (FBS)	Gibco, USA	12657-029
hexamethyldisilazane	Sigma-Aldrich, USA	440191-1L
hydrogen peroxide	Molekular Química, Brazil	L0032-P1L
*n*-propyl gallate	Sigma-Aldrich, USA	P3130-100G
paraformaldehyde	Sigma-Aldrich, USA	30525-89-4
penicillin/streptomycin	Gibco, USA	15140-122
phalloidin	Sigma-Aldrich, USA	49409
phosphate-buffered saline	Sigma-Aldrich, USA	806552
resazurin powder	Sigma-Aldrich, USA	R7017
retinoic acid	Sigma-Aldrich, USA	R2625
Triton X-100	Sigma-Aldrich, USA	9002-93-1
trypsin	Gibco, USA	15090046
plastics and other nonperishable materials		
cell culture flasks (25 cm^2^)	Nest, China	707003
cell culture plates (6-well)	Mevalabs, Denmark	MVL-1010000
cell culture plates (24-well)	Kasvi, Brazil	K12-024
hive-patterned array	BioEdTech, Brazil	Bio3DStamp version 6
equipment and software		
CO_2_ incubator	Panasonic, Japan	MCO-170AICUV-PA
confocal laser scanning microscope	Leica, Germany	TCS SP8
GraphPad Prism 5.0	GraphPad Inc., USA	Prism 5.0
ImageJ Fiji	National Institutes of Health, USA	https://imagej.net/ij/
microplate reader	Bmg Labtech, Germany	VANTAstar
microscope (inverted fluorescent and phase contrast)	Olympus, USA	IX51 TH4-100
OriginPro 9	OriginLab Corp., USA	OriginPro9
scanning electron microscope	Zeiss, USA	EVO MA-10

### 2.3 Cell Culture

SH-SY5Y (ATCC-CRL-2266, cells from human neuroblastoma) were kindly provided by the Laboratory of Neurobiology at the Universidade Federal de São Paulo. Cells were cultivated in 25 cm^2^ cell culture flasks with the following complete medium: Dulbecco’s modified Eagle’s medium/nutrient mixture F12 (DMEM/F12) supplemented with 10% fetal bovine serum (FBS) and 1% penicillin/streptomycin at 37 °C in an atmosphere of 5% CO_2_. When 90% confluence was reached, cells were trypsinized and distributed for the following 2D or 3D assays.

### 2.4 2D models of AD

#### 2.4.1 Neuron-like Differentiation

SH-SY5Y cells were seeded at the density of 1 × 10^5^ cells/well (in a 24-well plate) and cultured in the complete medium for 24 h. After that, cells were cultured for 3 days in the following neuronal phenotype differentiation medium: DMEM/F12 supplemented with 0.5% FBS, 10 μM retinoic acid (RA), and 1% penicillin/streptomycin.

#### 2.4.2 Oxidative Stress Induction

To determine the optimal concentration for inducing oxidative stress, on the fourth day of cultivation (1 day in the complete medium followed by 3 days in the neuron differentiation medium), cells were incubated with different concentrations of H_2_O_2_ (0 to 200 μM), for 1 h, diluted in the neuronal differentiation medium. The group exposed to 100 μM H_2_O_2_ was named “mild oxidative stress”, while the one exposed to 200 μM H_2_O_2_ was considered under “severe oxidative stress”. The following two-dimensional assays were performed right after H_2_O_2_ exposure.

#### 2.4.3 Cell Viability

The resazurin assay was used to assess the cell viability. Briefly, the medium was substituted with 10% resazurin solution diluted in the neuronal differentiation medium. Cells were incubated at 37 °C and 5% CO_2_ for 2 h. Subsequently, the solution of each well was transferred to a new plate for fluorescence measurement using a microplate reader (bottom-reading; excitation: 544 nm; emission: 590 nm). As a negative control, a cell-free 10% resazurin solution was incubated for the same period under the same conditions. The results are presented as a percentage of the control.

#### 2.4.4 Cell Population

Cells were fixed with 4% paraformaldehyde (PFA, diluted in phosphate-buffered solution, PBS), stained with DAPI, and imaged by a fluorescent microscope. Individual nuclei per 40× image were counted by using the ImageJ software.

#### 2.4.5 Reactive Oxygen Species (ROS) Production

To examine ROS, cells were incubated with the CellROX orange reagent (5 μM diluted in the neuronal differentiation medium) for 30 min at 37 °C and 5% CO_2_. Stained cells were imaged using a confocal laser scanning microscope (excitation 545/28 nm and emission 565/40 nm wavelengths). Fluorescence intensity quantification was performed using the ImageJ software.

Also, to evaluate ROS and to compare with the CellROX assay, cells were incubated with 2′,7′-dichlorodihydrofluorescein diacetate (DCFDA, 40 μM, diluted in neuronal differentiation medium) for 45 min at 37 °C and 5% CO_2_. Then, fluorescence was measured using a microplate reader (excitation: 485 nm; emission: 535 nm). The results are presented as percentage of the control.

#### 2.4.6 Congo Red (CR) Binding Assay

The CR dye binding assay was used to monitor the deposition of Aβ aggregates within the groups. Cells were fixed in 4% PFA and incubated in 0.5% CR (w/v, diluted in PBS) for 30 min. Cells were washed with PBS three times, incubated with phalloidin and DAPI for another 30 min, and washed twice, and slides were mounted with *n*-propyl gallate.

Cells were imaged by a phase contrast microscope and a confocal laser scanning microscope. For the phase contrast images, Aβ aggregates were quantified as the percentage of stained images. For the images obtained by a confocal microscope, the following particle analyses were performed.

#### 2.4.7 Aβ Particle Analyses

For the groups stained with CR and imaged with confocal microscopy, the following characteristics of Aβ aggregates were evaluated (Table 2). Using the particle analyzer tool of the ImageJ software, imaged samples were thresholded and spatially calibrated. Particle size was set from 0 to 10,000,000 nm^2^ to exclude anything that was not an object of interest in the image.

**Table 2:** Aβ Aggregate Analyzed Parameters

Aβ aggregate analyses	
parameter	function
circularity	The circularity measures how close to a circle the shape of an object is. It is calculated as a function of the area and perimeter using the following equation: $\mathrm{circularity}=\frac{4\times \pi \times \mathrm{area}}{{\mathrm{perimeter}}^{2}}$ . Circularity ranges from 0 to 1. A circularity value of 1.0 indicates a perfect circle. As the value approaches 0, it indicates an increasingly elongated polygon.
perimeter	the length of the outside boundary of the particles, in nm
total area	the total area of particles per image, in nm^2^
major diameter	the longest diameter (axis length) of each particle
minor diameter	the shortest diameter (axis length) of each particle
solidity	The solidity indicates how smooth and convex the particle contour is, according to the following equation: $\mathrm{solidity}=\frac{\mathrm{area}}{\mathrm{convex}\;\mathrm{area}}$ . The solidity ranges from 0 to 1. Particles with a dented contour with many cavities will have a low solidity close to 0. A perfectly convex particle will have a value of 1. First, the convex hull of the object is determined, and then the solidity is computed.
average size	the average area of individual particles per image, in nm^2^
number of aggregates	the number of particles per image

#### 2.4.8 Neurite and Cytoskeleton Distribution and Morphological Aspects

To evaluate the neurite distribution of differentiated SH-SY5Y, cells were fixed in 4% PFA (for 20 min, at room temperature) and permeabilized with 0.1% Triton X-100 (diluted in PBS) for 15 min. After three washes in PBS, cells were incubated with phalloidin (1:1500), PBS, and DAPI for 30 min at room temperature and imaged by a confocal laser scanning microscope.

#### 2.4.9 Scanning Electron Microscopy (SEM)

To evaluate morphological cellular aspects, cells were fixed in 4% PFA and dehydrated with a crescent sequence of acetone (30, 50, 70, and 100%) for 5 min in each solution. After that, they were immersed in acetone/hexamethyldisilazane (HMDS, 1:1) for 5 min and finally in HMDS until the solution evaporated. Samples were dried, sputter-coated with a thin layer of gold, and observed under a scanning electron microscope.

### 2.5 3D Spheroid Model of AD

#### 2.5.1 Preparation of Hydrogel Microwells

Agarose hydrogel microwells were fabricated to produce 3D multicellular neuronal spheroids. Briefly, 1.5 mL of warm autoclaved 2% agarose solution (w/v, diluted in PBS) was poured onto the wells of a six-well plate and stamped with a rigid resin hive-patterned array, producing uniform-sized microwells. The plate was placed on the top of an ice pack to accelerate the gelation process of the stamped hydrogel. Ten to 15 min later, the stamps were removed, and microwells in the culture plate were rinsed with PBS and kept under UV light for at least 30 min. After that, the plates were sealed and stored at 4 °C until cells were seeded onto them.

#### 2.5.2 Scanning Electron Microscopy (SEM)

The surface observation of the microwells was performed by SEM. Hydrogels were dehydrated and prepared for SEM analysis, as described in Section 2.4.9.

#### 2.5.3 Spheroid Production and Neuron-like Differentiation

SH-SY5Y cells were seeded at a density of 5 × 10^5^ cells/microwells-stamped well and cultured in the complete medium for 5 days. After that, cells were cultured for another 5 days in the neuronal phenotype differentiation medium (as described in Section 2.4.1). Both complete and differentiation media were changed every 2–3 days.

#### 2.5.4 Oxidative Stress Induction

On the 10th day of cultivation (5 days in the complete medium followed by 5 days in the neuron differentiation medium), spheroids were exposed to 200 μM H_2_O_2_ for 1h.

#### 2.5.5 Spheroid Area, Circularity, and Solidity

On a phase contrast microscope, spheroids were imaged on days 0–10 after cell seeding on microwells. The area, circularity, and solidity of spheroids were quantified using the ImageJ software (as described in Table 2).

#### 2.5.6 Neurite and Cytoskeleton Distribution and Morphological Aspects

To evaluate the neurite distribution along the spheroids, after 4 or 10 days of cultivation, they were fixed in 4% PFA (for 40 min, at room temperature) and permeabilized with 0.1% Triton X-100 (diluted in PBS) for 15 min. After two washes in PBS, cells were incubated with phalloidin (1:500), PBS, and DAPI overnight at 4 °C in the dark. They were washed again twice in PBS and imaged with a confocal laser scanning microscope.

Also, to evaluate morphological aspects, fixed spheroids on the microwells were imaged by SEM, as described in Section 2.4.9.

#### 2.5.7 Spheroid Cytoviability

After oxidative stress induction as described in Section 2.5.4, spheroids were subjected to a cell viability assay as described in Section 2.4.3.

### 2.6 Statistical Analysis

Data are expressed as mean ± standard deviation. Statistical significance between groups was evaluated using *t* test or one-way analysis of variance (ANOVA) followed by Tukey post hoc test for multiple comparisons using GraphPad Prism 5.0. Statistical significance was set at *p < 0.05, **p < 0.01, ***p < 0.001, and ****p < 0.0001. Graphs were plotted in OriginPro 9.

## 3 Results and Discussion

Oxidative stress plays a pivotal role in AD. ROS exacerbates neurodegeneration by interacting with mitochondria, leading to oxidative damage,^22,23^ especially in neurons, which have high polyunsaturated fatty acid in membranes, high oxygen consumption, and weak antioxidant defense.^24^

Physiologically, endogenous antioxidants contribute to maintaining ROS (produced by mitochondria) at low levels; however, mitochondrial misfunction and neural inflammation disturb the redox balance.^25^ In AD, the main responsible misfolded protein, Aβ, aggregates and triggers inflammatory response in the brain, overproducing ROS, compromising energy supply, and consequently inducing oxygen stress,^23,24,26^ which, in turn, contributes with accumulation and deposition of Aβ.

In other words, oxidative stress and Aβ aggregates represent a self-perpetuating, synergetic, and deleterious cycle in AD. This vicious cycle is characterized by the interdependence and mutual reinforcement of both processes, with each event precipitating and exacerbating the other, and together, they contribute to the progression of the disease. This study is based on these both pillars, focusing on (i) demonstrating cellular events and Aβ particle characteristics of neuronal cells under mild and severe oxidative stress and (ii) producing a 3D spheroidic model of neuronal cells under oxidative stress.

### 3.1 2D Models of AD

In the 2D models, neuron-like cells were cultured and exposed to H_2_O_2_ under mild (100 μM) or severe oxidative stress (200 μM) conditions for 1 h. Before establishing these concentrations, the proliferation rates of SH-SY5Y cells in both undifferentiated and neuronally differentiated states were evaluated, confirming that neuronal differentiation significantly suppressed cell proliferation (Figure S1A). Following this validation, differentiated SH-SY5Y cells were exposed to increasing concentrations of H_2_O_2_ (ranging from 0 to 1600 μM) to determine their susceptibility to oxidative stress (Figure S1B). According to the literature, SH-SY5Y cells differentiated into neuronal phenotype also demonstrated damage, reduced viability, and impaired cell size when exposed to 100 μM H_2_O_2_ for 1 h.^27^

As illustrated in Figure 1A, cytoviability statistically decreased when cells were exposed to mild or severe oxidative stress, representing 65.28 (±10.32) and 56.71 (±4.06%) of the control group, respectively (*p* < 0.0001, compared to the control group). The cell count in both the control and severe oxidative stress groups was also quantified (Figure 1B), showing similar numbers with no statistical difference between them. Collectively, Figure 1A,B demonstrated that, although the total number of cells remained constant after 1 h of H_2_O_2_ exposure, their viability was markedly reduced as a consequence of the oxidative damage.

**Figure 1:**
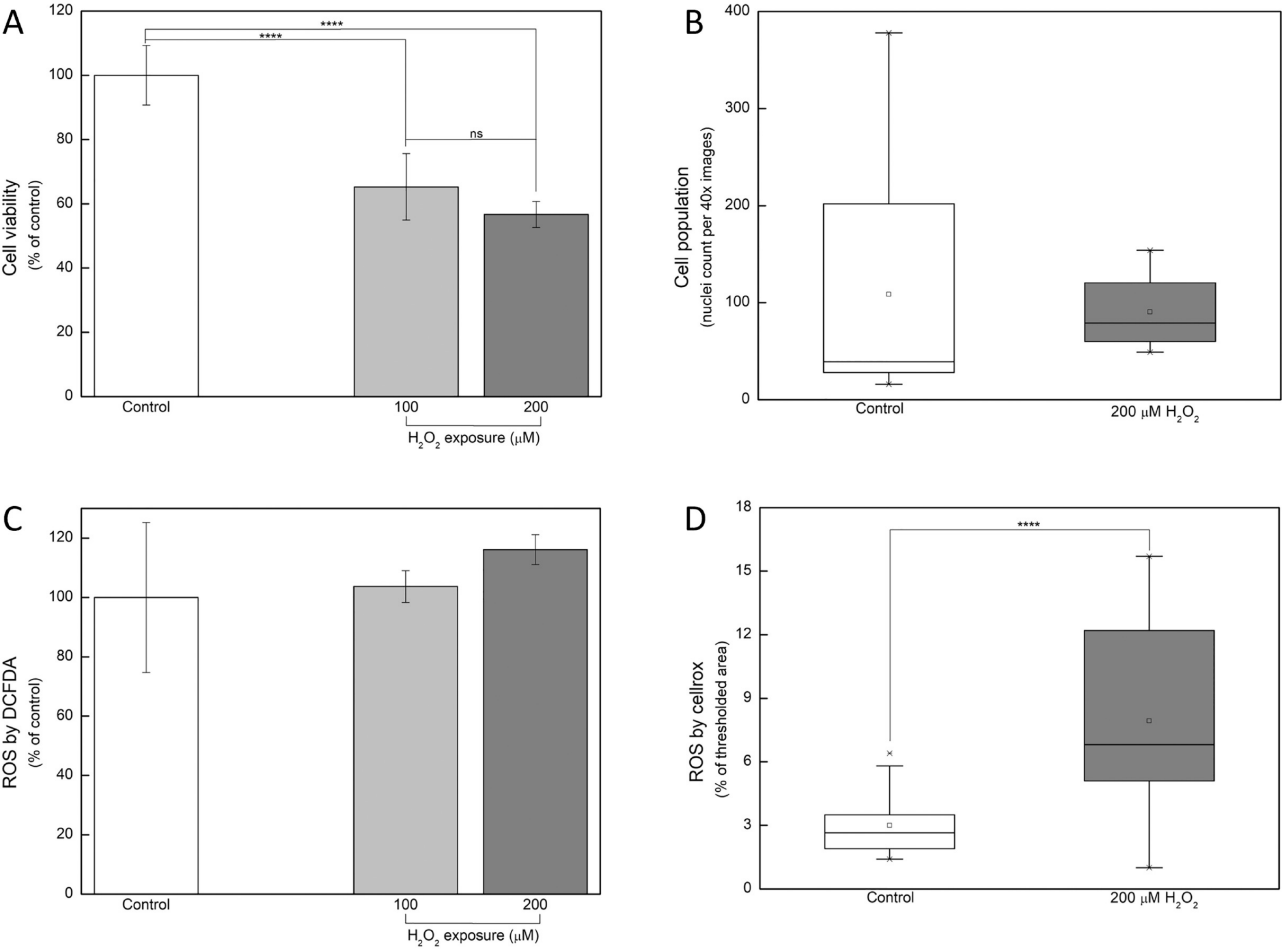
Oxygen peroxide (H_2_O_2_) modulates SH-SY5Y differentiated into neuron-like cell viability and reactive oxygen species (ROS) under mild (100 μM) or severe (200 μM) exposure. (A) Cell viability is equally decreased by both mild and severe H_2_O_2_ exposure. (B) Cell population is not statistically altered by severe H_2_O_2_ exposure. (C) ROS production assessed by DCFDA assay. (D) ROS staining quantification by CellROX assay (ns: nonsignificative and **** *p* < 0.0001, compared between indicated groups).

One of the most frequently employed oxidative stress inducers, H_2_O_2_, generates ROS that overwhelms cellular antioxidant defenses, resulting in protein damage and aggregation. H_2_O_2_ is a non-free-radical ROS that can penetrate cellular membranes, acting as an extra-mitochondrial effector. If not tightly regulated, both free-radical and non-free-radical ROS cause oxidative damage to mitochondrial proteins, lipids, and DNA, leading to potentially fatal consequences.

Regarding ROS, by the DCFDA assay (Figure 1C), no statistical differences were observed between groups; however, this can be attributed to the reduced viability of cells under stress, as shown in Figure 1A. To validate this hypothesis, live cells were stained with CellROX, imaged, and quantified for ROS (Figure 1D). There was a substantial difference between the evaluated groups, confirming that the absence of a statistical difference in the DCFDA assay is likely a consequence of the low cytoviability induced by the stress condition.

Groups were then stained for Aβ aggregates using CR, one of the principal methods used for detecting the Aβ structure of protein aggregates.^28^ CR is a diazo dye that binds selectively to β-sheet-rich aggregates, such as Aβ structures, through hydrophobic interactions and hydrogen bonding.^29^ This binding provides valuable information regarding the aggregation state of Aβ proteins, primarily targeting fibrils but also binding oligomers and protofibrils. Although CR is extensively utilized in histochemistry, its application in cellular models remains limited.

As a byproduct of the physiological breakdown of the amyloid precursor protein (APP), under physiological conditions, Aβ plays a role in the normal function of neuronal cells.^30,31^ As shown in Figure 2, the control group and the one under mild oxidative stress exhibited very few Aβ particles with no statistical difference between the percentage of these two groups. However, as an AD hallmark, there is a considerable accumulation of Aβ aggregates under severe oxidative stress, which contributes to neuronal dysfunction and cell death. In fact, Aβ itself can even generate ROS.^8,32^ On the other hand, oxidative stress can also impair the clearance of Aβ, increasing its concentration inside the brain and contributing significantly to AD onset and progression.^8^

**Figure 2:**
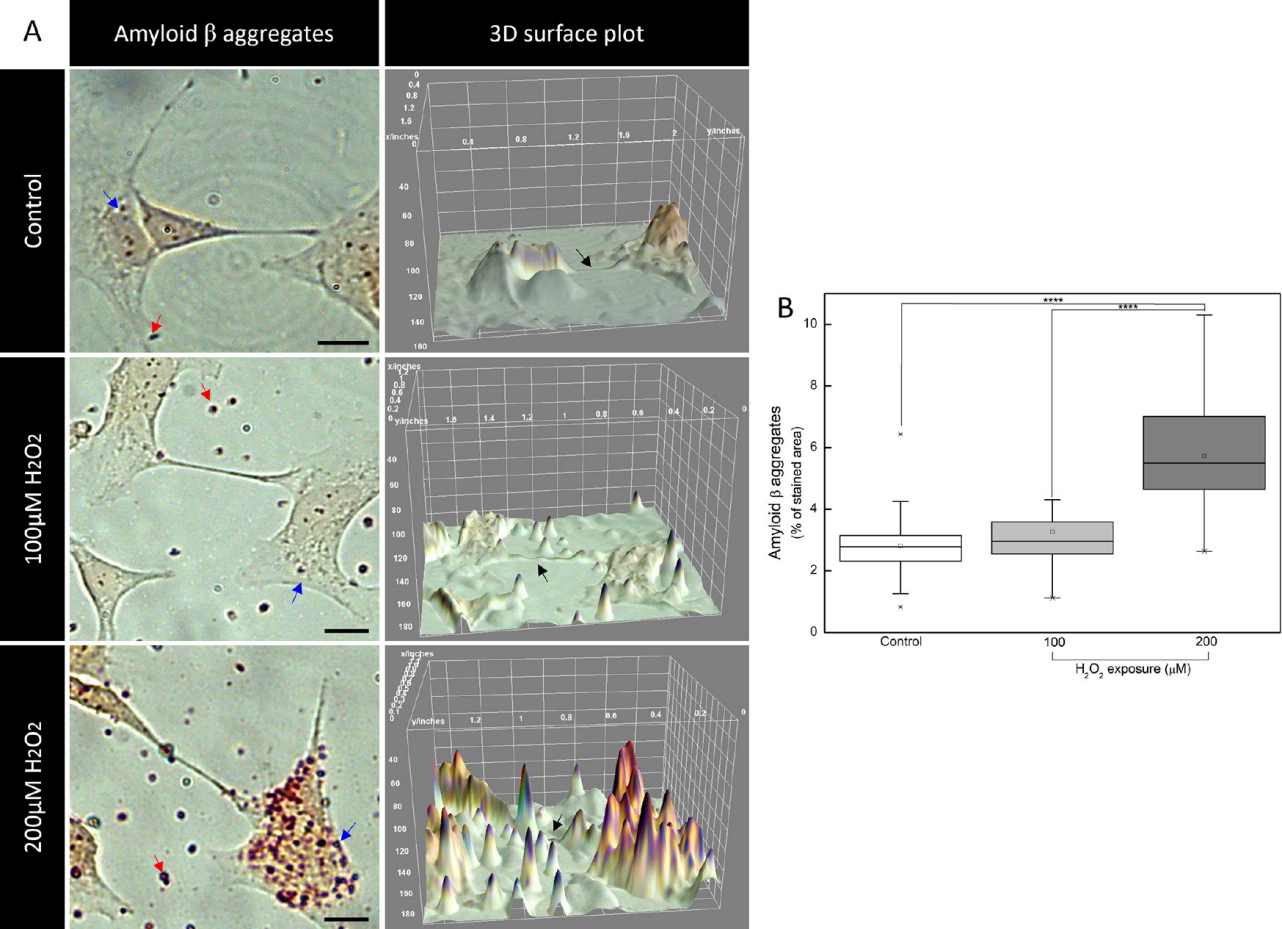
Severe H_2_O_2_ exposure induces Aβ aggregation in neuron-like cells. (A) Micrographs and 3D surface plots of Aβ aggregates stained by Congo red. Blue arrows indicate intracellular Aβ aggregates, while red arrows point to the extracellular ones and black arrows point to neurite 3D reconstitution. Scale bars: 10 μm. (B) Quantification of stained areas of Aβ aggregates (****p < 0.0001, compared between indicated groups).

In Figure 2A,B, under severe oxidative stress, the distribution of Aβ aggregates, both inter- and intracellularly, was significantly increased (*p* < 0.0001, compared with control or with mild stress group). These findings provide valuable insights about the cellular interplay involving the mechanisms between Aβ and oxidative-stress-related neurodegeneration. Highly oxidatively stressed groups can mimic late-stage AD pathophysiology, while the one under mild stress resembles the initial stages of neuronal damage in AD. When images were plotted into a 3D surface, the neurite distribution of cells under both mild and severe stress was twisted and diffused, as indicated by the black arrows in Figure 2.

The role of Aβ properties in both healthy and impaired brains with AD has been extensively analyzed by Moffat et al. (2022)^33^ through a longitudinal study. They characterized early changes in Aβ accumulation and brain network function, advancing the understanding of Aβ staging and its impact on cognitive decline. The brain is highly vulnerable to oxidative damage due to its high oxygen consumption,^34^ abundant lipid content,^35^ and relatively low antioxidant defenses.^36^ Elevated levels of oxidative markers in AD patients suggest a strong link between oxidative stress and disease progression, potentially contributing to disease onset.^5^ Primary sources of oxidative stress in the brain include mitochondrial respiration and enzymatic reactions, which are increased in AD. Aβ oligomers also generate ROS,^32^ further exacerbating oxidative damage. Extensive Aβ plaque formation induces neuronal apoptosis, excitotoxicity, and endothelial dysfunction, contributing to neuroinflammation and cognitive decline.

Corroborating these findings described in this work, the properties of Aβ aggregates resulting from mild or severe oxidative stress were evaluated at an *in vitro* level. CR-stained groups were imaged using a laser confocal microscope (Figure 3), and various features of the Aβ aggregates were quantified (Figure 4A–H). Under fluorescence imaging (Figure 3), Aβ aggregates were significantly more abundant in the group subjected to severe oxidative stress, while the control and mild stress groups exhibited discrete presence of aggregates.

**Figure 3:**
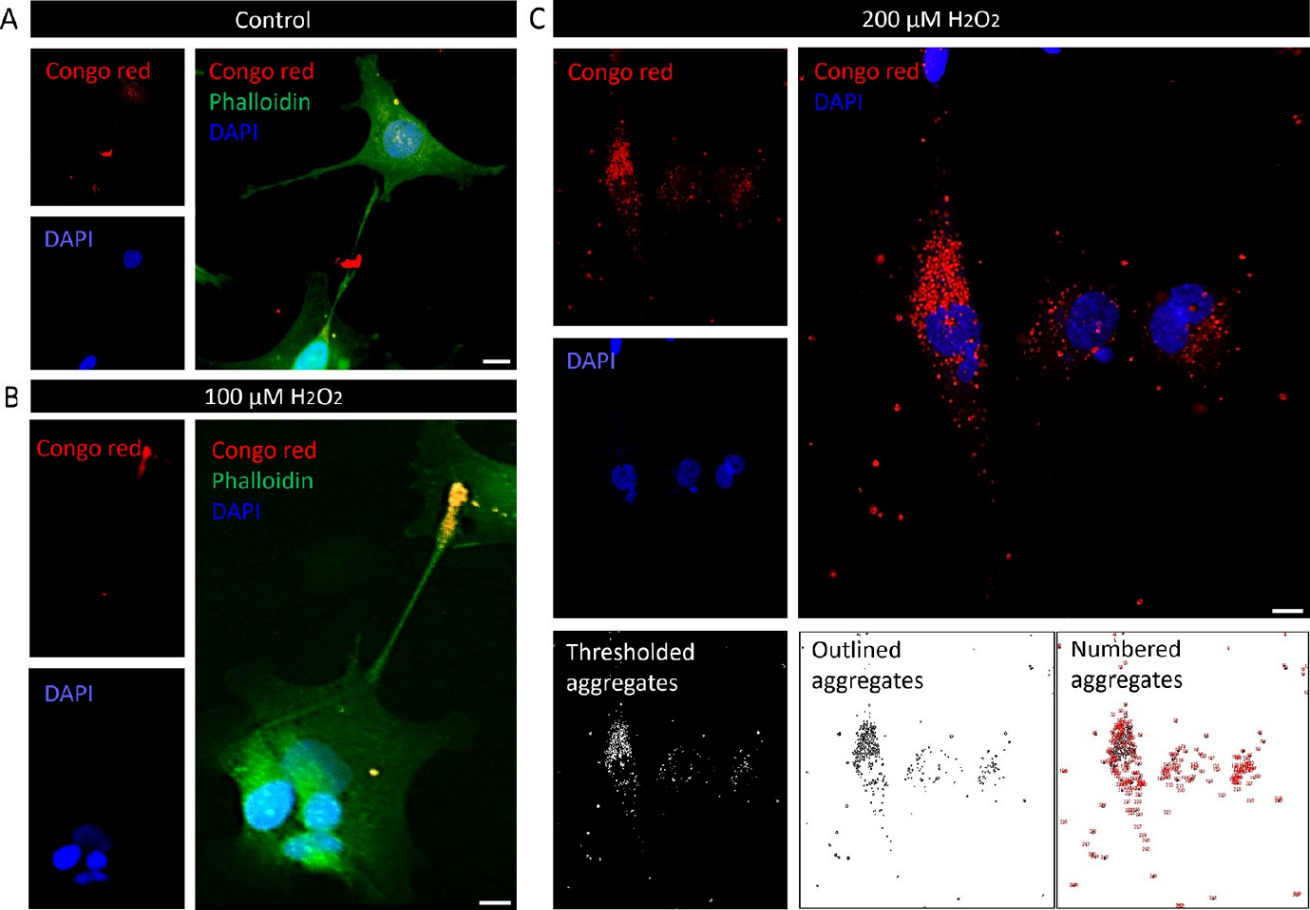
Aβ aggregates and cytoskeleton staining in SH-SY5Y under control, mild (100 μM), or severe (200 μM) H_2_O_2_ exposure. Congo red, phalloidin, and DAPI staining of (A) control, (B) mild, or (C) severe oxidative stress group followed by merged images and Aβ aggregates thresholded, outlined, and numbered for quantification. Scale bars: 10 μm.

**Figure 4:**
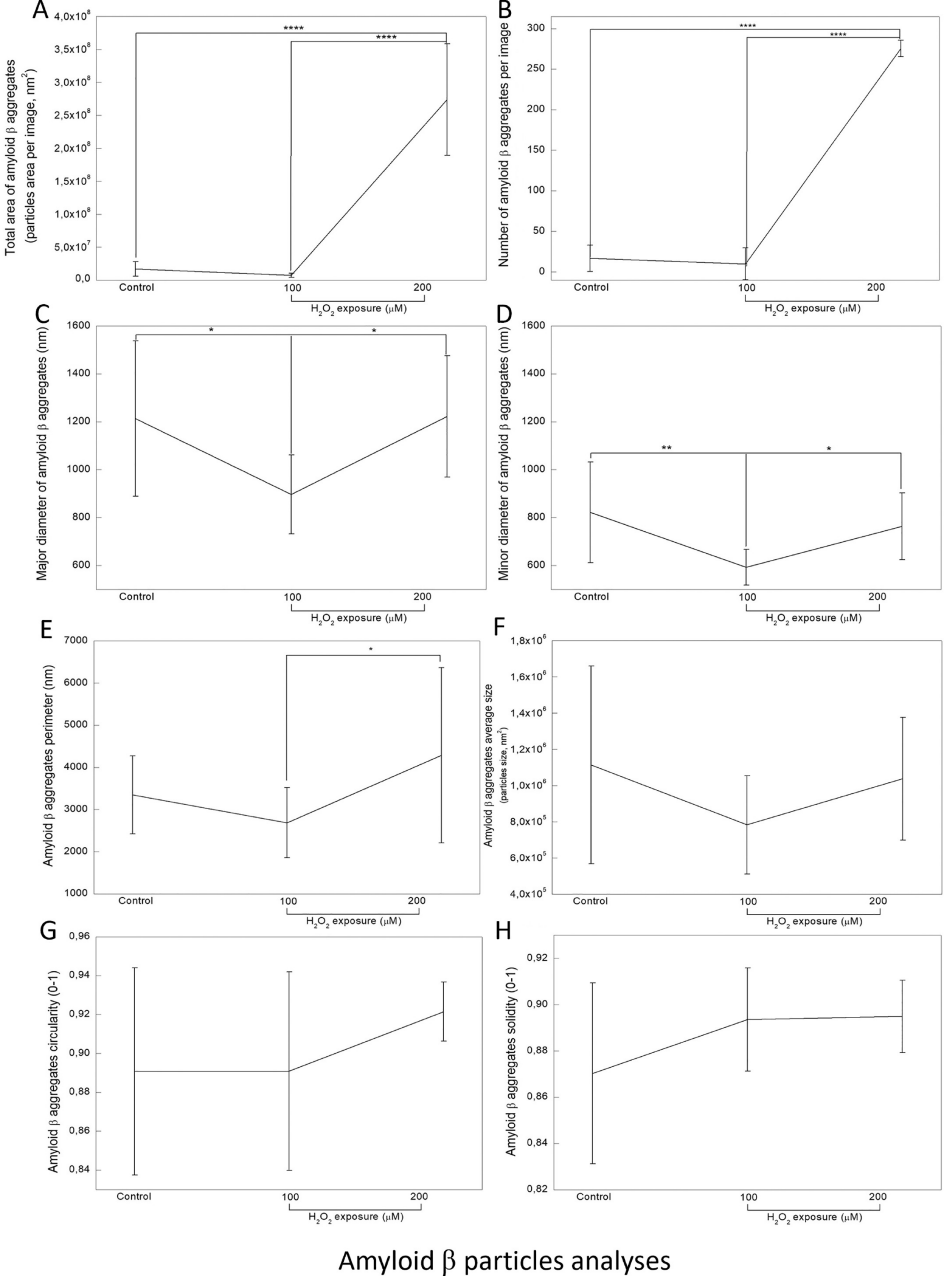
Aβ aggregate properties of differentiated SH-SY5Y cells under mild (100 μM) or severe (200 μM) H_2_O_2_ exposure. (A) Total area, (B) number per image, (C) major diameters, (D) minor diameters, (E) perimeter, (F) average size, (G) circularity, and (H) solidity of Aβ aggregates (* *p* < 0.5; ** *p* < 0.01 and **** *p* < 0.0001, compared between indicated groups).

In the detailed analysis of Aβ aggregates, the total area of Aβ staining (Figure 4A) and the number of aggregates per image (Figure 4B) statistically increased in cells under severe stress (*p* < 0.0001, compared with the control or mild stressed group). Although the control and mild stress groups had very few Aβ aggregates, it is noteworthy that the major (Figure 4C) and minor (Figure 4D) diameters of the observed aggregates were statistically smaller in the mild stress group than in the other two groups. The perimeter of aggregates was also significantly larger in the severe stressed group compared to the one under mild stress (*p* < 0.05, Figure 4E). Moreover, no statistical differences were observed between groups regarding the average size (Figure 4F), circularity (Figure 4G), and solidity (Figure 4H) of the Aβ aggregates.

Cells were then stained with phalloidin/DAPI and imaged by laser confocal microscopy or by SEM to observe the morphological aspects and neurite distribution under the three evaluated conditions (Figure 5). As observed, under no H_2_O_2_ exposure, neurites had a healthy spread-out morphology. Under severe stress, however, neurites were abnormally twisted and dystrophic, a hallmark of AD,^37^ representing a major contributor to synaptic dysfunction. The resultant neurite sprouting and profound cytoskeletal alterations may follow the chronic stimulation of the stereotypical reaction to both exacerbated ROS and Aβ aggregate accumulation. Aβ deposits alter microtubule dynamics,^38^ and as Aβ compacts more, forming oligomers, fibrils, and senile plaques, the synaptic function of neurons progressively fails in AD.^39,40^ The greater the Aβ burden is, the higher are the levels of ROS and synaptic loss, initially in the hippocampus and cerebral cortex, driving the progression of the disease and reaching distinct areas of the brain.

**Figure 5:**
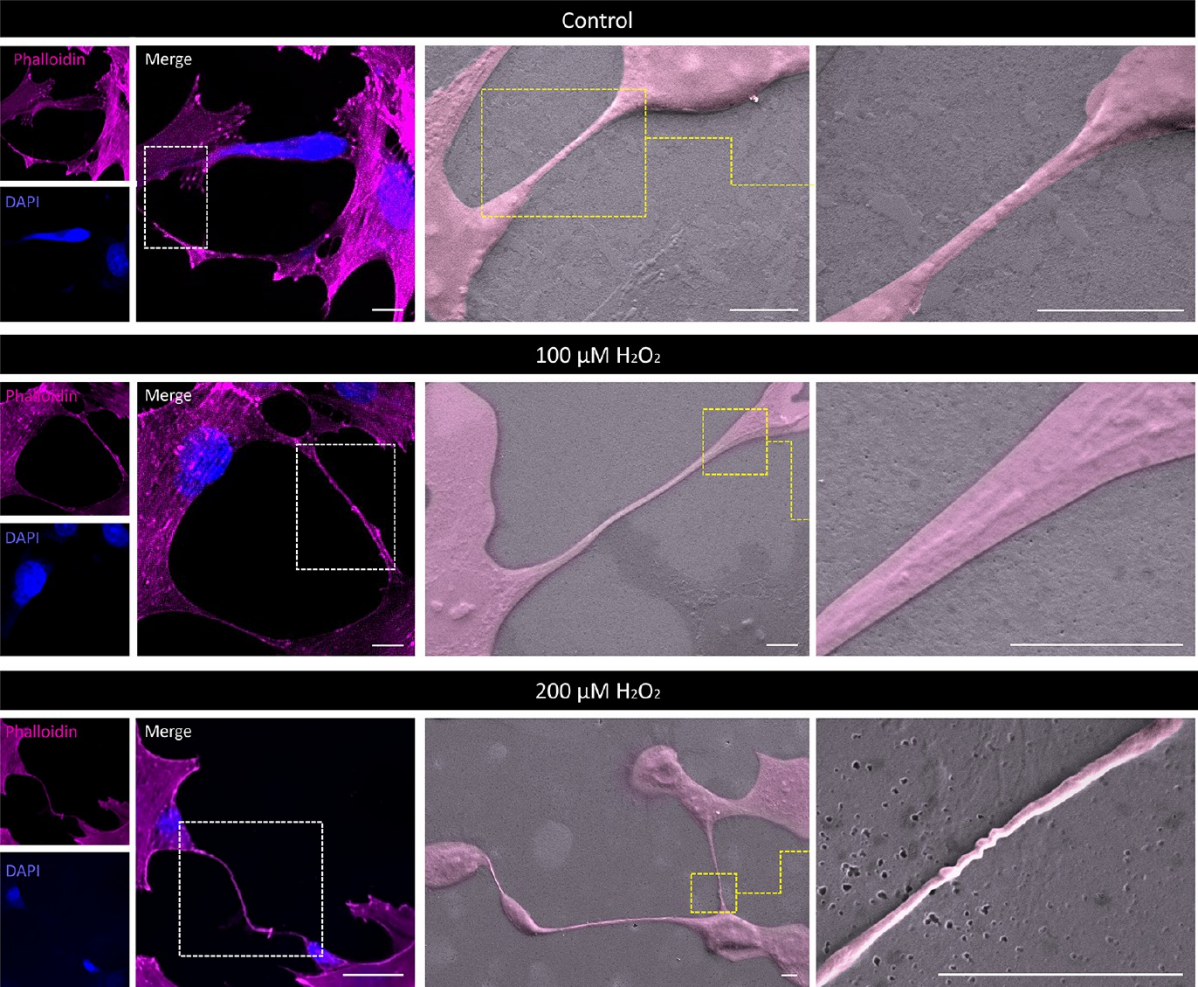
Morphological modulation of H_2_O_2_-induced stress on actin cytoskeleton neurites of differentiated SH-SY5Y. Phalloidin and DAPI staining followed by SEM micrographs of SH-SY5Y differentiated cells under control, mild (100 μM), or severe (200 μM) H_2_O_2_ exposure. Oxidative stress induced by severe H_2_O_2_ exposure modulates the structural integrity of the actin cytoskeleton neurites, showing a dystrophic and twisted morphology. White delimited areas highlight the morphology of stained neurites, and yellow delimited areas indicate the zoomed areas of SEM images. Scale bars: 10 μm.

Seidel et al. (2012)^41^ described, in a tauopathy 3D model of neurodegeneration with SH-SY5Y cells, the distinction of neurite retraction versus massive cell death, outlining tau mutation-dependent cellular degeneration and depicting a progression from an intact neuronal structure under physiological conditions to cell swelling, neurite retraction, degeneration, severe retraction, and ultimately cell death under tauopathy. Neurite retraction is initiated by microtubule instability, leading to the breakdown of the neuronal network and disintegration of the spheroidal structure.^42^

Moreover, corroborating the observation of twisted neurites under stress (Figure 5), a study from the early 2000s^43^ reported that the capacity of Aβ plaques to cause neuronal damage is closely linked to their structural characteristics. According to this study, the cytoskeletal distribution in dystrophic neurite formation changes from preclinical to end-stage AD, and the authors even suggest that the progression to dementia was associated with both a shift to a higher proportion of fibrillar plaques that induced local neuritic alterations and a transformation of cytoskeletal proteins within associated abnormal neuronal processes. Additionally, a recent study by Tsering et al. (2025)^44^ with human *post mortem* brains described that neuritic dystrophy surrounding Aβ plaques induced the clustering of microglia (the immunity cells of the brain) as well as a phenotypic shift in a brain region-specific manner, modulating the neuroimmune response and, consequently the neuroinflammation in AD. These findings suggest that damaged dystrophic neurites around Aβ aggregates may act as triggers for recruiting microglia to the Aβ deposited region, contributing to the interplay between neuroinflammation and neurodegeneration, regarding the importance of neurite dystrophy.^10,45^

In addition, another recent study by Wyatt-Johnson et al. (2025)^46^ emphasized the role of the MR1/mucosal-associated invariant T (MAIT) cell axis in the development of AD, identifying MR1 as a crucial component of the axis in dystrophic neurite formation. The MR1/MAIT cell axis is part of the innate immune system, involving the interaction of the major histocompatibility complex (MHC) class I-related molecule MR1 with MAIT cells. This axis is associated with a number of other inflammatory disorders, indicating that the investigation of innate immune alterations and their impact on the development of dystrophic neurites and Aβ aggregates can enhance the knowledge of AD pathogenesis and potentially lead to the identification of novel therapeutic targets for the disease.

### 3.2 3D Models of AD

Modern neurobiology science has increasingly focused on reducing animal use, avoiding counterspecies models, and improving *in vitro* models, thereby contributing to the principle of the 3R: replacement, reduction, and refinement. With this purpose, modeling AD three dimensionally is particularly important, as the pathology involves complex interactions between brain cells and intra- and extracellular proteins and components that are challenging to replicate in flat, monolayer cultures. Supporting this goal, various studies have also dedicated their efforts to developing 3D models using SH-SY5Y, focusing on Parkinson’s disease,^47^ tauopathy,^41^ cocultured systems with endothelial cells,^48^ bioprinting AD models,^49^ investigating the interaction between electromagnetic fields and biological systems,^50^ evaluating cytotoxic agents,^51^ and addressing a novel differentiation protocol.^52^

For this reason, to enrich the quality of our 2D models of AD and to address the limitations of traditional 2D cell cultures, SH-SY5Y spheroids were three-dimensionally self-assembled by cultivation on developed biocompatible nonadhesive microwells, with complete medium culture (for 5 days), followed by a neuron-like differentiation medium for an additional 5 days. On the 10th day of cultivation, spheroids were exposed or not to H_2_O_2_ to simulate the classic neurotoxic event of oxidative stress observed in AD.

As shown in Figure 6A,B, the hydrogel-based microwells exhibited homogeneity, robustness, and resistance to degradation, facilitating *in situ* cell aggregation. The precise depth and dimensions of the patterned microwells effectively prevented spheroid–spheroid fusion, thereby enhancing cell seeding efficiency. Furthermore, the uniform spatial arrangement of the spheroids within the same spatial plane significantly facilitated imaging acquisition.

**Figure 6:**
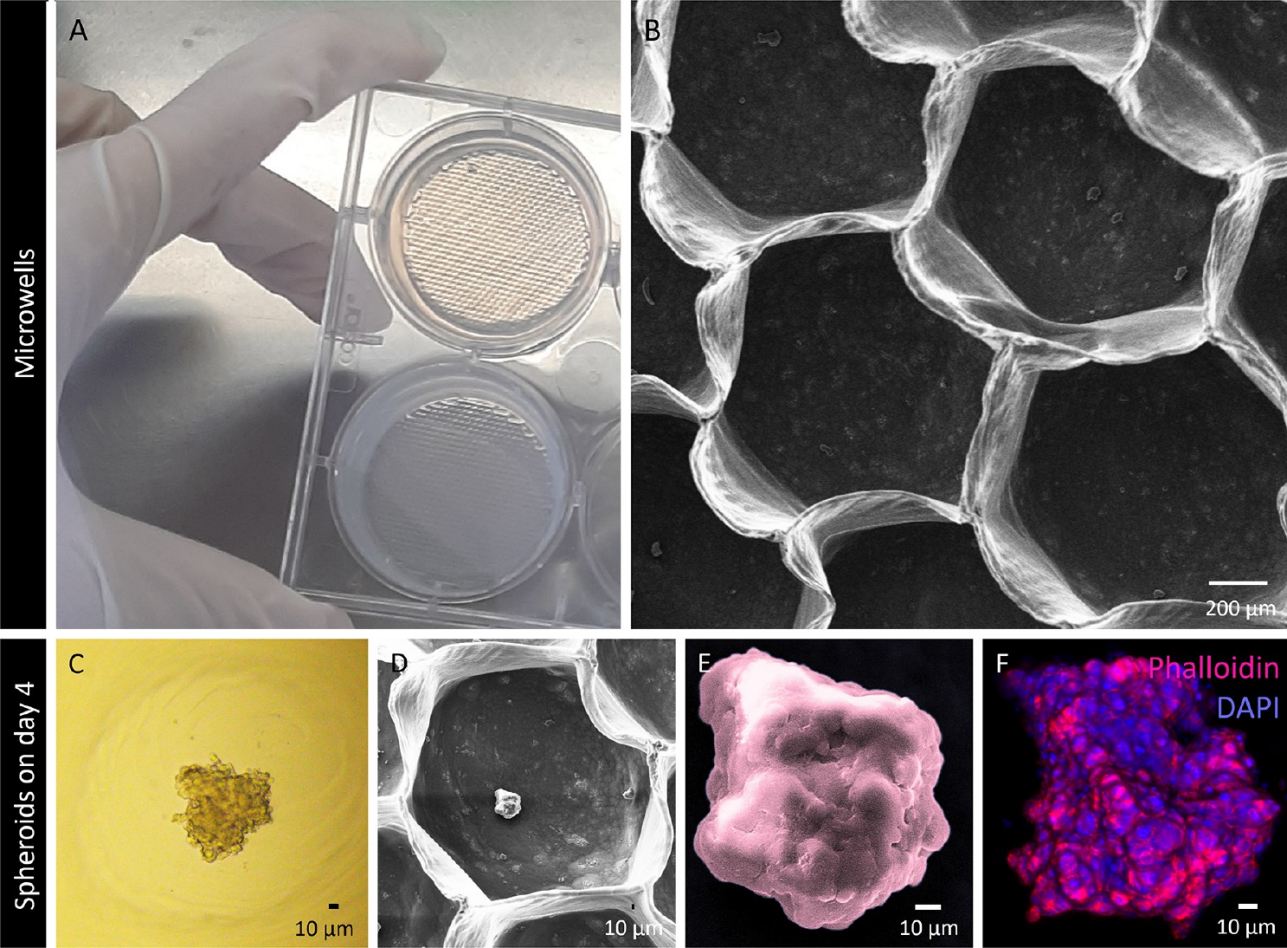
Microwells and 3D spheroid production and characterization. (A) Macroscopic view of the microplate with microwells for free-float spheroid culture. (B) SEM micrograph of the microwells. (C, D) Brightfield micrographs showing the SH-SY5Y differentiated neuronal model in microwells for 4 days. (E) SEM micrograph providing a detailed view of the spheroid on day 4. (F) Z-stack imaging of the cytoskeleton (phalloidin, magenta) and nucleus (DAPI, blue) staining of the spheroid on day 4.

In comparison with another fundamental widely used technique for 3D modeling, bioprinting, our self-assembled spheroids had the primordial advantage of not being immersed in a polymeric matrix, which can limit cellular spread and complicate the extraction of cells and cellular components. Although bioprinting allows for the creation of more complex brain environment scenarios,^18,20,53,54^ the technique of producing spheroids on nonadherent microwells offers several benefits: simplicity, cost-effectiveness, scalability, and ability to produce uniform, high-quality spheroids with minimal cellular. In contrast, bioprinted cells may experience high stress when passing through the printer needle and during the extrusion process. Moreover, the selection of cross-linking agents in bioprinting must be meticulously considered to prevent compromising cellular integrity and viability.^55^ For instance, calcium chloride (CaCl_2_) can depolarize neurons under a prolonged exposure, and thermoresponsive or light-responsive cross-linking can result in temperature fluctuations or ultraviolet (UV) light exposure, which can permanently modulate, damage, or eventually kill bioprinted cells.

After 4 days of cultivation, as shown in Figure 6C–F, the spheroids exhibited distinct morphological characteristics. At this stage, they had undergone substantial self-assembly organization, transitioning from loose cell aggregates into more compact 3D structures, resulting in a dense conformation (Figure 6C–E). This compactness indicated robust cell–cell interactions (Figure 6F).

De Simone et al. (2018)^56^ developed spheroids with SH-SY5Y, seeding cells at a density of 5 × 10^4^ cells/mL. The growth of these spheroids was monitored over a period of 30 days without inducing neuron-phenotype differentiation. For their assay, spheroids at 5 days of cultivation were selected, as their size met the essential criteria for gradients of oxygen, nutrients, and proliferation rate required for a biorelevant spheroid screen;^57^ also, spheroids of larger size could potentially develop a necrotic core due to inadequate oxygen and nutrient exchange to the inner cells.

We established a protocol for cultivating spheroids involving 5 days in the complete medium followed by 5 days under neuron-phenotype differentiation. The morphological characteristics of spheroid formation were assessed over this 10 day period. Periodic imaging (Figure 7A) revealed that the spheroids progressively became more arranged, organized, rounded, and solid. By day 10 (Figure 7B), spheroids displayed advanced maturation and neuronal differentiation, indicating their progression into more complex and functional 3D structures compared to earlier stages (Figures 6C–F and 7A).

**Figure 7:**
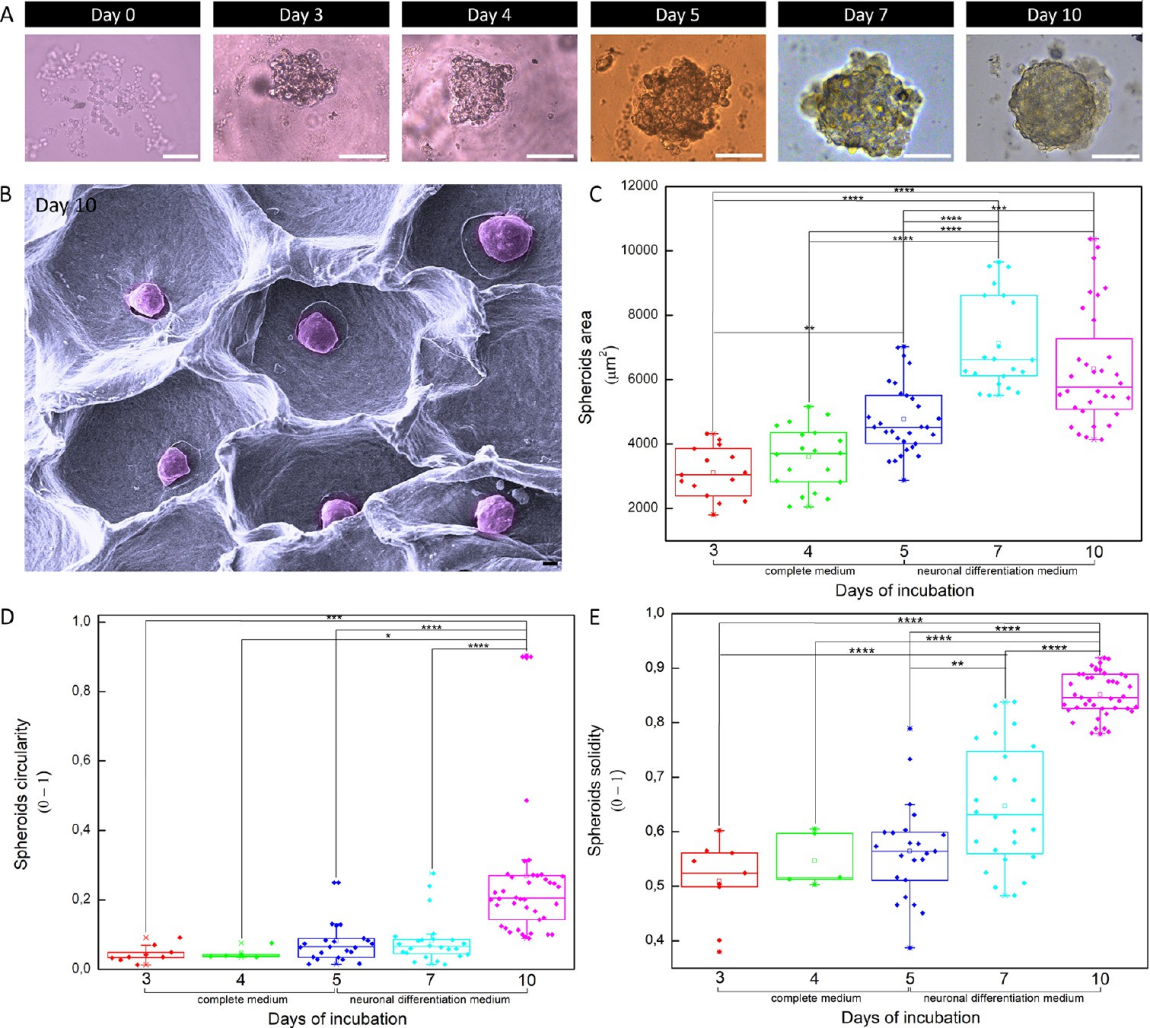
Spheroid morphology and growth metrics along the 10 days of development. (A) Brightfield micrographs of spheroids at different days of arrangement (days 0 to 10). (B) SEM micrograph of spheroids on day 10 of production. Spheroid (C) area, (D) circularity, and (E) solidity over the 10 days of organization in which the cells were incubated for 5 days under the complete medium followed by 5 days under neuronal differentiation one. Scale bar: 50 μm.

As demonstrated in Figure 7C, the area of the spheroids had a significant increase up to day 7. However, there was no statistical difference between the spheroid area on day 7 (7128 ± 1940 μm^2^) and day 10 (6341 ± 1789 μm^2^), suggesting that the differentiation medium may have reduced cell proliferation, promoting the induction of the neuronal phenotype. Notably, in terms of circularity (Figure 7D) and solidity (Figure 7E), spheroids on day 10 were statistically more rounded and robust, respectively, compared to the earlier time points (days 3 to 7). This indicates that although cell proliferation had reduced since day 7, the cellular organization of the spheroids continued to improve until day 10.

On day 10, the 3D models exhibited extensive neurite distribution throughout the spheroid’s composition (Figure 8A). Neurites extend from cells within the spheroid, predominantly on the surface, forming a network-like structure, with a solid and homogeneous shape, characterized by robust cell–cell interactions potentially mediated by adhesion molecules and gap junctions as well as strong cell–extracellular matrix interactions. These interactions are crucial for maintaining the structural integrity of the spheroids and supporting neuronal differentiation.

**Figure 8:**
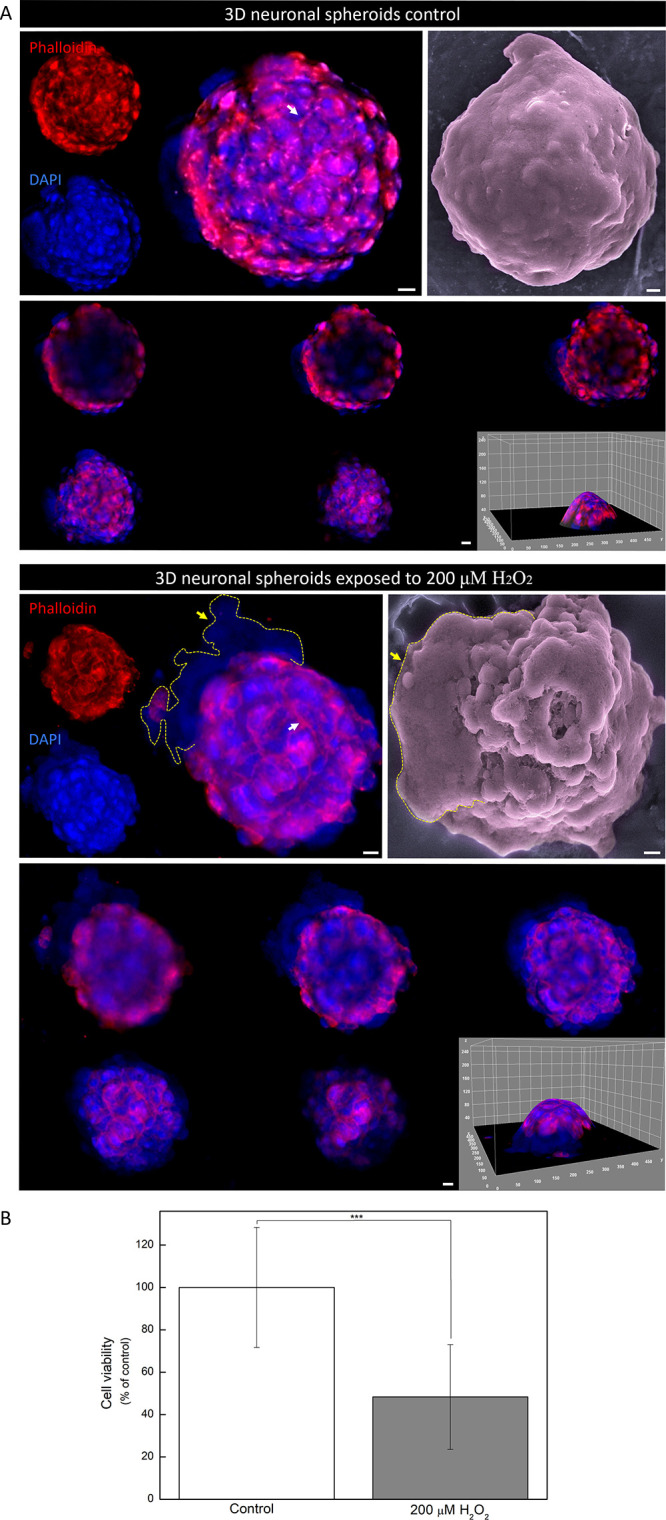
Neuronal spheroids exposed or not to H_2_O_2_. (A) Z-stack, followed by SEM micrograph, montage of stained Z-projection and 3D-plot reconstitution of representative neuronal differentiated SH-SY5Y spheroids stained with phalloidin/DAPI exposed or not to 200 μM H_2_O_2_, respectively. White arrows demonstrate neurite distribution, and yellow delimited area and arrow indicate nuclei disruption in the group under oxidative stress. Scale bars: 10 μm. (B) Cell viability is decreased by 200 μM H_2_O_2_ exposure (***p = 0.0004).

Also on day 10, spheroids were subjected to oxidative stress by exposure to 200 μM H_2_O_2_ (Figure 8A). Abnormal nuclear staining was observed surrounding the spheroids as well as in SEM micrographs, indicating atypical organization and nuclear envelope disruption. Morphological disarrangement of spheroids was also evident after H_2_O_2_ exposure, highlighting the importance of further studies regarding AD neurodegenerative pathways. H_2_O_2_ is known to induce DNA damage through free-radical mechanisms, and the presence of DNA fragmentation suggests selective and specific cleavage. This phenomenon is consistent with findings from the early 1990s, when Hinshaw et al. (1993)^58^ demonstrated that H_2_O_2_-induced oxidative stress in PC12 cell cultures led to membrane blebbing and neurite destruction. Similarly, Whittemore et al. (1995)^59^ reported that H_2_O_2_-induced apoptotic cell death in primary neuronal cultures resulted in cell blebbing, fragmented nuclei, and a DNA ladder of oligonucleosome-length fragments after 3 h of H_2_O_2_ exposure, which is similar to our observation 1 h postexposure in the 3D model. Moreover, still under 200 μM H_2_O_2_ exposure, cell viability within the spheroid (Figure 8B) statistically decreased to 48.33 ± 24.74% of the control group (*p* = 0.0004). In summary, the neuronal spheroids produced in this study represent a promising 3D model for elucidating complex cellular dynamics and pathways involved in AD.

Future research should focus on refining and validating both 2D and 3D models of AD to better replicate the intricate dynamics of oxidative stress. Incorporating proteins, such as tau and Aβ, and enhancing the spheroids with a diverse composition of cell types, such as glial and/or endothelial cells, are within the scope of our future modeling efforts. Ultimately, leveraging 3D models has the potential to accelerate the screening of effective therapeutic agents and enhance our understanding of AD pathogenesis and its associated pathways.

## 4 Conclusions

This study highlighted the impact of oxidative stress on neuron-like cells in both 2D monolayer culture and 3D spheroid models to validate their use as experimental platforms for AD. While 2D models provided an elementary and accessible system for investigating Aβ modulation and neurite imbalance in response to dose-dependent H_2_O_2_-induced oxidative stress, 3D models more accurately mimicked the *in vivo* neuronal microenvironment, exhibiting enhanced cellular interaction and oxidative-stress-induced damage, emphasizing the importance of integrating advanced 3D culture systems into AD research.

## Supplementary Material





## Notes

The resazurin assay was performed to verify, in the 2D model, if SH-SY5Y proliferation was reduced by the neuronal differentiation protocol (Figure 1A) and to determine SH-SY5Y cytoviability under distinct H_2_O_2_-concentrations exposure (Figure 1B) (PDF)
Confirmation of publication and licensing rights—openaccess (PDF)
