# Supplementary material for: 2D and 3D Models of Alzheimer’s Disease: Investigating Neuron-like Cells in Oxidative Environments
Source: ACS Omega. 2025 Jun 16;10(25):27501–14. doi: 10.1021/acsomega.5c03306 (PMC12223811; doi:10.1021/acsomega.5c03306)
Supplement: Supplementary file 1 [file ao5c03306_si_001.pdf]

# 2D AND 3D MODELS OF ALZHEIMER'S DISEASE: INVESTIGATING NEURON-LIKE CELLS IN OXIDATIVE ENVIRONMENTS

*Geisa R. Salles[1#], Luiza A. Giraldi[1], Newton S. da Silva[2], Marimelia A. Porcionatto[3,4], Cristina Pacheco-Soares[1#]*

## Supplementary file

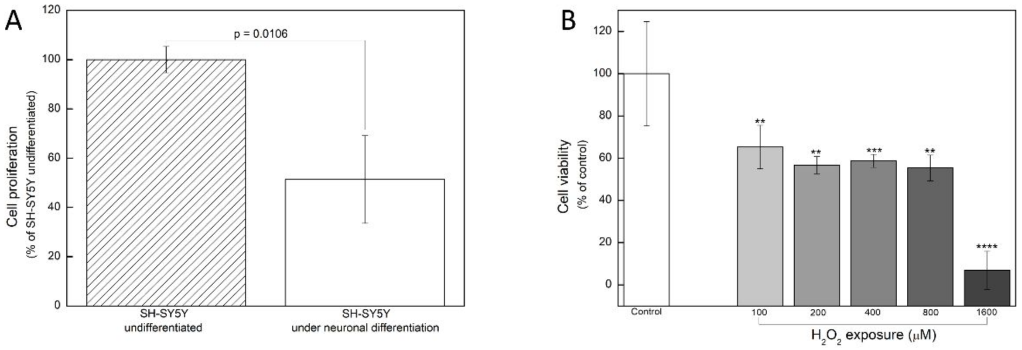


**Supplementary Figure 1:** *Neuronal differentiation and oxygen peroxide ($H_2O_2$) modulate SH-SY5Y cells.* (**A**) Cellular proliferation is decreased by neuronal differentiation protocol. (B) Viability of SH-SY5Y differentiated is statistically decreased by different concentrations (0-1600 μM) of $H_2O_2$ exposure. (** $p < 0.01$, *** $p < 0.001$ and **** $p < 0.0001$, compared to the control group, which represents SH-SY5Y differentiated into neuronal phenotype).
